# Mining the *Sinorhizobium meliloti* Transportome to Develop FRET Biosensors for Sugars, Dicarboxylates and Cyclic Polyols

**DOI:** 10.1371/journal.pone.0043578

**Published:** 2012-09-24

**Authors:** Alexandre Bourdès, Steven Rudder, Alison K. East, Philip S. Poole

**Affiliations:** Department of Molecular Microbiology, John Innes Centre, Norwich, United Kingdom; University of Florida, United States of America

## Abstract

**Background:**

Förster resonance energy transfer (FRET) biosensors are powerful tools to detect biologically important ligands in real time. Currently FRET bisosensors are available for twenty-two compounds distributed in eight classes of chemicals (two pentoses, two hexoses, two disaccharides, four amino acids, one nucleobase, two nucleotides, six ions and three phytoestrogens). To expand the number of available FRET biosensors we used the induction profile of the *Sinorhizobium meliloti* transportome to systematically screen for new FRET biosensors.

**Methodology/Principal Findings:**

Two new vectors were developed for cloning genes for solute-binding proteins (SBPs) between those encoding FRET partner fluorescent proteins. In addition to a vector with the widely used cyan and yellow fluorescent protein FRET partners, we developed a vector using orange (mOrange2) and red fluorescent protein (mKate2) FRET partners. From the sixty-nine SBPs tested, seven gave a detectable FRET signal change on binding substrate, resulting in biosensors for D-quinic acid, *myo-*inositol, L-rhamnose, L-fucose, **β**-diglucosides (cellobiose and gentiobiose), D-galactose and C4-dicarboxylates (malate, succinate, oxaloacetate and fumarate). To our knowledge, we describe the first two FRET biosensor constructs based on SBPs from Tripartite ATP-independent periplasmic (TRAP) transport systems.

**Conclusions/Significance:**

FRET based on orange (mOrange2) and red fluorescent protein (mKate2) partners allows the use of longer wavelength light, enabling deeper penetration of samples at lower energy and increased resolution with reduced back-ground auto-fluorescence. The FRET biosensors described in this paper for four new classes of compounds; (i) cyclic polyols, (ii) L-deoxy sugars, (iii) β-linked disaccharides and (iv) C4-dicarboxylates could be developed to study metabolism *in vivo*.

## Introduction

Bacteria have evolved a wide variety of metabolic strategies to cope with varied environments. Some are specialists adapted to restricted environments, while others are generalists that cope with diverse environmental conditions. Rhizobia (e.g. *Rhizobium, Sinorhizobium, Bradyrhizobium, Mesorhizobium* and *Azorhizobium* species) can not only survive and compete for nutrients in soil and the plant rhizosphere but can also form beneficial symbioses with legumes in highly specialized plant cells located in root nodules. The number of SBP-dependent transporters in rhizobia is much higher than in the majority of other bacteria so far sequenced. This may be due to the need for the acquisition of a broad range of growth-limiting nutrients from soil, rhizosphere and during symbiosis, facilitated by the high affinity of SBPs for specific solutes [1], [2], [3], [4].

The ATP-binding cassette (ABC) superfamily is a large ubiquitous group of transporters that possess a common minimum structure consisting of four domains: two hydrophobic integral membrane domains and two ATP-binding domains. A subfamily of ABC transporters found exclusively in prokaryotes can be distinguished from other ABC transporters by the presence of a SBP responsible for the uptake of solutes. This SBP is located in the periplasm of Gram-negative bacteria and attached to the cell membrane in Gram-positive bacteria and Archaea [5], [6]. SBPs also function with secondary transporters that utilise the electrochemical gradient across the cytoplasmic membrane to energise the transmembrane movement of solutes. The known SBP-dependent secondary transporters are currently placed in two families, the TRAP transporters and a smaller family of tripartite tricarboxylate transporters (TTT). Both families have a similar tripartite structure comprising an SBP and two unequally sized integral membrane components, but have unrelated amino acid sequences [7], [8].

Transcriptional induction of these transporters was previously studied by creating a suite of plasmid and integrated fusions to nearly all ABC and TRAP transporters of *Sinorhizobium meliloti* [9]. Putative promoter regions for ABC and TRAP transporter operons were cloned upstream of reporter genes. In total, nearly 500 fusions were made and these were tested with 174 inducing conditions. Overall, specific inducers were identified for 76 transport systems, amounting to 47% of the ABC uptake systems and 53% of the TRAP transporters in *S. meliloti*. Of these transport systems, 64 were previously uncharacterized in rhizobia and 24 were induced by solutes not known to be transported by ABC- or TRAP-uptake systems in any organism [9]. We showed that specific solutes from wide range of compounds; mono- and oligosaccharides, polyols, amino acids, amines and organic acids, act as transcriptional inducers thus providing a global expression map of the transportome of *S. meliloti* [9].

While induction biosensors, based on following gene expression by means of a monitored marker protein, are relatively easy to develop and reliable in the environment, they are of limited use to monitor flux *in vivo*. This is for two major reasons; (i) they require their bacterial host to grow, (ii) growth involves a substantial lag in detection time and resolution. In contrast, protein based biosensors, by their very nature, are able to interact with other molecules, including small ligands, binding them rapidly with great specificity. When this binding is accompanied by a conformational change in protein structure, distinction between unbound protein and bound complex is possible. A conformational change on ligand binding can be linked to a direct detection method e.g. optical, electrochemical or mechanical.

In this study we aimed to develop real-time biosensors relying on FRET, in which the change in transfer of fluorescent energy between fluorophores is analysed. It requires an overlap between emission and excitation spectra of a suitable donor/acceptor pairs. The Főrster distance (*R*_0_) defines the distance at which transfer is 50% efficient between a pair of fluorophores, and is dependent upon their spectral overlap, the relative orientation of the chromophore transition dipoles and the quantum yield of the donor in the absence of the acceptor. As *R*_0_ is usually between 2 and 6 nm (in the range of protein dimensions), FRET has been used as a ‘microscopic ruler’ within approx.1–10 nm range [10]. FRET can be used as a highly sensitive indicator of protein conformational change as it is a non-destructive spectroscopic method of optically monitoring relative orientation of fluorophores and distance apart [11]. FRET biosensors are suitable for both *in vitro* and *in vivo* detection of ligands and have been used for real-time monitoring of metabolites in various cells and cellular compartments [12], [13], [14], [15], [16], [17], [18]. The range of analytes detectable by FRET biosensors is currently twenty-two compounds distributed in eight classes of chemicals (two pentoses, two hexoses, two disaccharides, four amino acids, one nucleobase, two nucleotides, six ions and three phytoestrogens) [19], [20].

In this study, newly developed FRET vectors were used to clone SBPs with defined expression profiles from the transportomes of *S. meliloti* and, the closely related bacterium, *Rhizobium leguminosarum*, together with the SBP from a single well-characterised dicarboxylate transporter from *Rhodobacter capsulatus* [21], [22], [23]. From the generally applicable assumption that transcriptional inducers of ABC-uptake systems bind to the corresponding SBP, we have developed seven FRET biosensors to detect cyclic polyols, L-deoxy sugars, β-linked disaccharides and C4-dicarboxylates.

## Results And Discussion

### Development of FRET vectors to clone core SBPs

**Figure 1:**
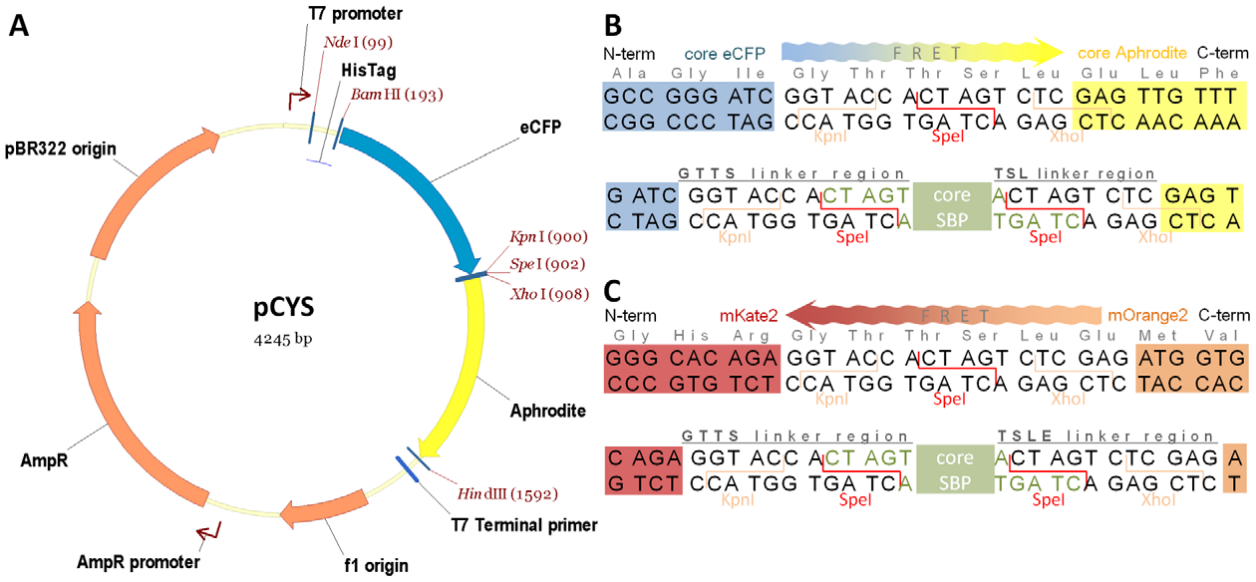
FRET vector and insertion sites for cloning SBPs. (**A**) vector pCYS and cloning sites of (**B**) pCYS and (**C**) pROS. Direction of energy transfer between fluorescent proteins is shown. Fluorescent proteins are coloured coded; N-terminal His-tagged-eCFP (blue), Aphrodite (yellow), N-terminal His-tagged-mKate2 (red), and mOrange2 (orange). The position of core SBP insertion (green) is shown, together with the amino acid sequences of N-terminal (GTTS) and C-terminal (TSL/TSLE) linkers.

Our aim in this project was to screen all SBPs for which we had characterised a promoter induction profile to see if any could form the basis of a FRET biosensor. For *S. meliloti* data came from a comprehensive study using reporter fusions [9], while for *R. leguminosarum*, data was available from microarray experiments [9], [24]. It was considered that transcriptional inducers would, in most cases, be a ligand of the cognate SBP which could then be screened for a FRET signal change on interaction with that ligand. The gateway-compatible FRET vector pGWF1 is available for the cloning of genes encoding SBPs between two fluorescent protein partners (N-terminal cyan fluorescent protein (CFP) and C-terminal yellow fluorescent protein (YFP)) but this creates long linkers of 20 and 21 amino acids between the fluorescent proteins and SBP [21], [22], [23]. A detailed study on linker length showed that a shorter linker length usually improves the FRET ratio change upon binding of ligand [11], [25]. Therefore, we devised a new high-throughput cloning vector pCYS with short linkers (Fig. 1A). The strategy we devised relies on cloning PCR products using BD In-Fusion PCR cloning by using the unique SpeI site for fragment insertion (Fig. 1). This powerful strategy of ligation-independent cloning is unaffected by the presence of a SpeI site in the insert.

In order to broaden the range of applications for which FRET biosensors could be used, a vector based on different fluorescent proteins was developed. Using FRET between red fluorescent protein (RFP) and orange fluorescent protein (OFP) allows the use of longer wavelength light, enabling deeper penetration of samples at lower energy and increased resolution with reduced back-ground auto-fluorescence. In addition, these proteins are not based on modification of green fluorescent protein (as CFP, YFP and all their variants are) but have their origins in corals (mOrange2 is a highly photostable variant of a wavelength-shifted monomeric derivative of DsRed from *Discoma sp.*) [26] and sea anemonies (mKate2, a brighter variant of mKate which combines pH resistant and photostability with a far-red emission spectrum) [27]. As an intermediate in construction of pROS (a vector based on FRET between RFP and OFP), a plasmid pLMB634 (Table S1) was made. This plasmid has RFP (mKate2) and YFP (Aphrodite) cloned either side of SMc02324 which binds L-rhamnose. It was hoped that FRET between mKate2 and Aphrodite could be detected, but initial results with pLMB634 showed that this was not the case.

The FRET vector pCYS was tested by inserting the glucose binding protein from *Escherichia coli* (MglB) [11]. This SBP was reported to have a FRET Δ ratio ranging from −8% to −28% upon binding of glucose that depended on linker length [11]. The purified eCFP-MglB-Aphrodite fusion protein from pLMB240 (Table S1) gave a FRET Δ ratio of −19%, thus validating this vector and the choice of short linkers (of four and three amino acids) which we had incorporated into its design (Fig. 1B).

Following validation of pCYS vector, the next step was to clone SBP genes with defined induction profiles. From over a hundred SBP genes for which induction profiles are known [9], [24], sixty-nine were selected for further study because of the potential interest of their putative ligands. Of these, fifty-eight were from *S. meliloti*, ten were from *R. leguminosarum* and one from *R. capsulatus* (Table S2). All sixty-nine were successfully cloned in pCYS, in each case forming fusion proteins eCFP-coreSBP-Aphrodite (Fig. 1B).

To establish that each of the sixty-nine purified fusion proteins displayed functional fluorescent domains, the N-terminal eCFP was excited at 433/10 nm and the emissions of both eCFP and the C-terminal Aphrodite (YFP) were measured at 475/10 nm and 525/10 nm respectively. The 525/475 nm FRET ratio in the absence of any added ligand (apo ratio) (Table S2, FRET ratio >0.7 (good or v. good)) was recorded before testing a range of different possible ligands. Forty constructs showed a FRET ratio >0.7 (Table S1), indicating that the two fluorophores are close to each other in the apo-form of the protein. If the FRET ratio was <0.6, a possible explanation is that the Aphrodite domain (YFP) was not functional, probably due to incorrect folding. This was tested by direct excitation (ex 510/6 nm; em 525/8 nm) (Table S1, YFP folding; classified as good or poor). For the great majority of the twenty-nine constructs for which we obtained a FRET ratio of <0.6, it could be explained by a lack of emission from YFP. However there were 10 constructs for which the YFP signal was present, but the FRET ratio was <0.6 (Table S1). It may be that in these the fluorescent proteins are too far away from each other for FRET to occur.

From the forty constructs with a FRET ratio >0.7, the intensity of the FRET signal changed (>1%) on ligand binding in seven of these constructs (Table 1). For those SBPs which gave <1% FRET signal change on ligand addition, it may be that the SBP is pre-bound with a ligand that has co-purified. In those cases there would be insignificant FRET change detected on ligand binding as the protein (or a significant proportion of it) is already in the bound conformation [28].

**Table 1:** Summary of FRET biosensors developed.

FRET biosensor	Detects	Family	Inducer of gene expression (fold change)^1^	Core SBP	Best structural model	Parent vector	Plasmid	ΔFRET on saturation (%)	Ligand bound	K_d_ (M)
SMb20036-CY	quinic acid	TRAP	D-quinic acid (8.7)	E27-	Class II/	pCYS	pLMB229	−4.7±0.4	D-quinic acid	5.9±1.1×10^−9^
				Q338	cluster E				shikimic acid	1.0±2.7×10^−4^
SMb20712-CY	*myo*-inositol	ABC;CUT2	*myo*-inositol (6.4)	E22-	Class I/	pCYS	pLMB319	4.7±0.7	*myo-*inositol	4.7±2.5×10^−7^
				N309	cluster B					
SMc02324-CY/	L-rhamnose	ABC;CUT2	L-rhamnose (>6.2)	E25-	Class I/	pCYS	pLMB291	−5.5±0.4	L-rhamnose	7.3±1.3×10^−9^
SMc02324-RO			erythritol (>6.2)	F329	cluster B				L-fucose	1.1±0.2×10^−5^
						pROS	pLMB523	6.4±0.5	L-rhamnose	6.9±1.4×10^−9^
									L-fucose	1.9±0.5×10^−5^
SMc02774-CY	L-fucose	ABC;CUT2	D-fucose (6.7)	Q33-	Class I/	pCYS	pLMB292	−4.2±0.4	L-fucose	3.6±1.7×10^−9^
			pyruvate (4.9)	N329	cluster B				L-rhamnose	1.6±0.6×10^−6^
			L-fucose (4.2)							
SMc04259-CY	β-diglucosides	ABC;CUT1	gentiobiose (19)	T24-	Class I/	pCYS	pLMB345	2.1±0.2	cellobiose	3.3±0.9×10^−8^
				D441	cluster B				gentiobiose	5.3±2.1×10^−8^
RL2376-CY	D-galactose	ABC;CUT2	D-arabinose (3.6)	A31-	Class I/	pCYS	pLMB394	5.2±0.4	D-galactose	5.3±1.7×10^−6^
			D-galactose (2.6)	Y327	cluster B					
rcc03024-CY	C4-	TRAP	NA	E27-	Class II/	pCYS	pLMB414	−2.7±0.2	succinate	3.6±0.7×10^−8^
	dicarboxylates			E333	cluster E				fumarate	5.8±1.2×10^−8^
									L-malate	7.0±1.4×10^−8^
									oxaloacetate	1.6±0.6×10^−5^

1 Inducer of gene expression is taken from [9]. NA: not applicable.

Ligands we were able to detect with FRET biosensors were (i) D-quinic acid (ii) *myo-*inositol, (iii) L-rhamnose, (iv) L-fucose, (v) β-diglucosides (cellobiose and gentiobiose), (vi) D-galactose and (vii) C4-dicarboxylic acids (Table 1). A biosensor has already been developed for the hexose D-galactose [19], but this work demonstrates FRET biosensors for four new classes of targets; cyclic polyols (D-quinic acid and *myo*-inositol), L-deoxy sugars (L-rhamnose and L-fucose), β-diglucosides and C4-dicarboxylates.

### FRET biosensors for cyclic polyols

Two of the biosensors detected cyclic polyols; D-quinic acid and *myo*-inositol. Both these compounds are commonly found in plants and the fact that SBPs from bacteria which live in close association with plants have formed the starting-point for biosensor development is not surprising.

**Figure 2:**
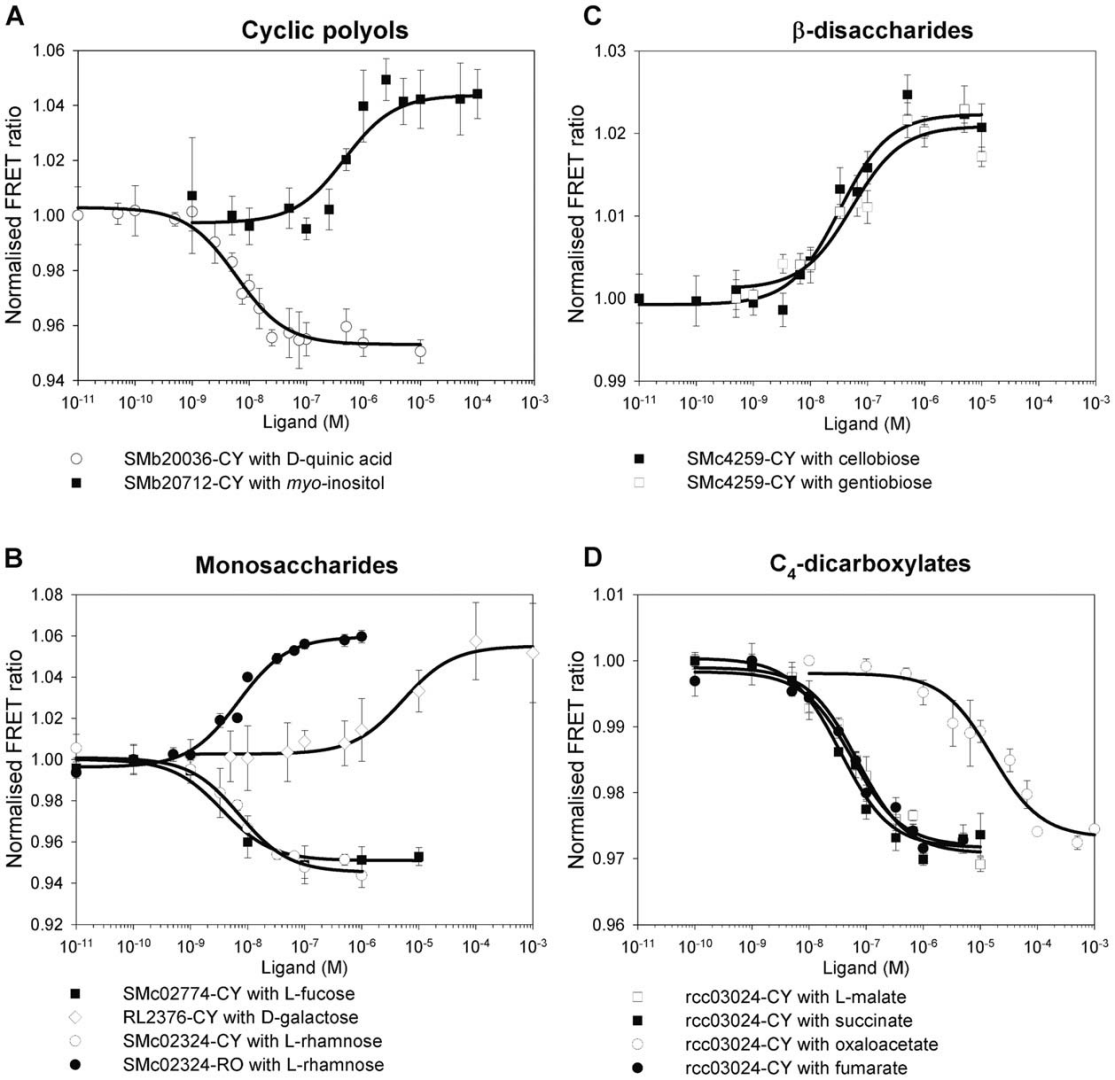
Normalised ligand binding isotherms for FRET biosensors. (**A**) cyclic polyols; SMb20036-CY binding D-quinic acid and SMb20712-CY binding *myo*-inositol, (**B**) monosaccharides; SMc02774-CY binding L-fucose, RL2376-CY binding D-galactose, SMc02324-CY and SMc02324-RO binding L-rhamnose, (**C**) disaccharides; SMc04259-CY binding cellobiose and gentiobiose, (**D**) C4- dicarboxylates; rcc03024-CY binding L-malate, succinate, oxaloacetate and fumarate. The biosensor is designated by the gene encoding the SBP protein core, followed by the fluorescent protein pair (where C is CFP (eCFP), Y is YFP (Aphrodite), R is RFP (mKate2) and O is OFP (mOrange2)). All data points are an average of at least two independent protein purifications tested on technical quadruplicates.

The biosensor for D-quinic acid, a component of bark and found in other plant-derived material, is based on the SBP of a TRAP-transporter, SMb20036. Previous results had shown that the gene encoding SMb20036 was induced 8.7-fold by D-quinic acid [9]. The TRAP transporter components are encoded by SMb20034-6, while SMb20037 is annotated as a shikimate 5-dehydrogenase (AroE). Protein purified from SMb20036 cloned in pCYS (SMb20036-CY) displayed an *in vitro* ligand concentration-dependent decrease in FRET ratio of -4.8±0.4% on binding D-quinic acid (K_d_ 5.9±1.1×10^−9^ M) and shikimic acid (K_d_ 1.0±2.7×10^−4^ M) (Table 1, Fig. 2A). Since there is a huge difference in the affinity for D-quinic acid compared with that for shikimic acid (>15,000-fold), this indicates that SMb20036 encodes a high-affinity D-quinic acid binding protein. Indeed, the change in FRET ratio on addition of shikimic acid may be due to low residual contamination with D-quinic acid. There was no change in FRET ratio with other compounds similar in structure to D-quinic acid; gallic acid, benzoic acid or *N*-acetyl-5-neuraminic acid (Neu5Ac) (sialic acid). A FBS-RO version was also made but although a FRET signal was displayed transferring energy from the excited C-terminal OFP to the N-terminal RFP, there was no change in FRET ratio on binding either D-quinic or shikimic acid. In the FBS-RO version the linker length is increased to four amino acids either side of the SBP insert (Fig. 1C), but it is not known whether this is significant, or if it is the different properties of the fluorescent proteins in this vector which lead to this result.

SWISS-MODEL threads the tertiary structure of core SMb20036 on SiaP (27% identity (id)) from *Haemophilus influenzae*, an extracytoplasmic solute receptor of sialic acid Neu5Ac (PDB: 3B50) [29], [30]. This predicts N- and C-termini occur on the same lobe, defining it as Class II [31] and cluster E SBP, as are all SBPs associated with TRAP systems grouped according to Berntsson's classification [32]. All TRAP-dependent SBPs structurally characterized so far have conserved features, such as a long α-helix and two large β-strands spanning the hinge region, one of which is part of the two five-stranded β-sheets found in each domain [32]. The amino acids forming hydrogen bonds with the carboxylic acid group of Neu5Ac in SiaP (Arg127, Arg147 and Asn187) are highly conserved and found in matching position in the core of SMb20036. It is assumed that these residues are important for binding between D-quinic acid and SMb20036.

The inositol biosensor is based on SMb20712, a SBP of an ABC-transporter of the carbohydrate uptake transporter (CUT) 2 sub-family, the gene of which was induced 6.4-fold by *myo*-inositol [9]. The ABC transporter operon (SMb20712 (SBP), SMb20713 (ATP binding cassette) and SMb20714 (permeases)) is preceded by SMb20711 which was identified as a putative inosose isomerase. A wide range of bacteria, including *R. leguminosarum* and *S. meliloti*, can catabolise *myo*-inositol, via inosose leading to malonic semialdehyde and acetyl-CoA feeding the TCA cycle [33], [34], [35]. The purified SMb20712-CY protein displayed an *in vitro* ligand concentration-dependent increase in FRET ratio of 4.7±0.7% upon binding to *myo*-inositol (K_d_ 4.7±2.5×10^−7^ M) (Table 1, Fig. 2A). SMb20712-CY did not react with either inositol 1,4,5-trisphosphate or phytic acid, which were tested as they have similar structures to inositol, but with phosphate groups replacing hydroxyl groups.

SWISS-MODEL threaded the tertiary structure of core SMb20712 on two ribose binding proteins (which show 55% id to each other). The first is a ribose SBP from *Thermoanaerobacter tengcongensis* (PDB: 2IOY; 34% id) [36] and the second from *E. coli* (PDB: 2DRI; 30% id) [37], and are classified as belonging to Class I [31] and cluster B [32]. Many amino acids of the two ribose SBPs forming hydrogen bonds with β-D-ribopyranose are highly conserved; notably five of them (Asn13; Arg90; Arg145; Asn197 and Gln244) are found in matching positions in the core of SMb20712, where they are assumed to be important for binding *myo*-inositol.

### FRET biosensors for L-deoxy sugars

The methyl pentoses (or 6-deoxy-hexoses) L-fucose and L-rhamnose have the same molecular formula (C_6_H_12_O_5_) but differ in their structures. L-fucose (equivalent to 6-deoxy-L-galactose) is found on *N*-linked glycans on the surface of plant cells while L-rhamnose (or 6-deoxy-L-mannose) is commonly bound to other sugars and can be isolated as glycosides from plants. Two biosensors were developed; one which responds primarily to L-rhamnose but also, with a lower response, to L-fucose, while the second has the opposite properties showing a greater response to L-fucose.

The L-rhamnose biosensor is based on SMc02324, the SBP of a CUT2 ABC-transporter. The gene encoding SMc02324 was induced more than 6.2-fold by both L-rhamnose and erythritol [9] (Table 1). The rhamnose uptake and catabolic operon (RhaIDRSTPQUK) in *R. leguminosarum* b.v. *trifolii*, important for competitiveness during nodule formation, is orthologous (52–78% id) to the single gene cluster SMc02321-5, SMc03000-3 in *S. meliloti* [38], [39]. Purified SMc02324-CY displayed an *in vitro* ligand concentration-dependent decrease in FRET ratio of −5.5±0.4% upon binding to L-rhamnose (K_d_ 7.3±1.3×10^−9^ M) and L-fucose (K_d_ 1.1±0.2×10^−5^ M). SMc02324-RO displayed a ligand concentration-dependent increase in FRET ratio of 6.4±0.5% upon binding to L-rhamnose (K_d_ 6.9±1.4×10^−9^ M) and L-fucose (K_d_ 1.9±0.5×10^−5^ M) (Table 1, Fig. 2B). It is interesting to note that these biosensors showed an increase in FRET ratio on binding to SMc02324-RO and a decrease on binding to SMc02324-CY (Table 1) suggesting that the two fusion proteins fold very differently from one another. SMc02324-CY did not react with deoxy-D-ribose, D-fucose, tagatose, or the sugar alcohols, erythritol and dulcitol. Erythritol is an example of a specific inducer compound not binding to the product of the induced gene. Furthermore, the three order-magnitude difference in affinity of SMc02324 for L-rhamnose compared to L-fucose indicates it encodes a high-affinity L-rhamnose binding protein.

SWISS-MODEL threads the tertiary structure of core SMc02324 on autoinducer-2 (AI-2) receptors LsrB from *Salmonella typhimurium* (PDB: 1TJY; 35% id) and *S. meliloti* (PDB: 3EJW; 32% id) which are specialised SBPs that bind the quorum sensing signal AI-2 (a furanosyl borate diester synthesised from 1-deoxy-3-dehydro-D-ribulose and boric acid) [40], [41]. The two LsrBs have highly similar sequence (75% id) and are reported to be in Class I [31] and cluster B, positioning the N- and C-termini in separate domains [32]. The amino acids of LsrBs forming hydrogen bonds with their substrate are highly conserved; notably three of them (Lys10; Asp92 and Gln142) are found in matching positions in the core of SMc02324 where it is thought they are important in binding L-rhamnose.

The L-fucose biosensor is based on SMc02774, an SBP of another CUT2 ABC-transporter. This gene was induced by D-fucose (6.7-fold), pyruvate (4.9-fold) and L-fucose (4.2-fold) [9]. SMc02775, contiguous with genes encoding the ABC transporter (SMc02772-4), was annotated as a putative L-fucose dehydrogenase [4], [42]. Purified from SMc02774-CY displayed an *in vitro* ligand concentration-dependent decrease in FRET ratio of −4.2±0.4% upon binding to L-fucose (K_d_ 3.6±1.7×10^−9^ M) and L-rhamnose (K_d_ 1.6±0.6×10^−6^ M) (Table 1, Fig. 2B). SMc02774-CY did not react with D-fucose and pyruvate, another example of specific inducer compounds not binding to the product of their induced gene. Furthermore, the three order-magnitude difference between the affinity of SMc02774-CY for L-fucose compared to its affinity for L-rhamnose indicates that SMc02774 encodes a high-affinity L-fucose binding protein.

Despite the structural similarity of the two L-deoxy sugar ligands (L-fucose and L-rhamnose).and the fact that both SBPs (SMc02324 and SMc02774) are in the CUT2 sub-family, they show only 25% id to each other. SWISS-MODEL threaded the tertiary structure of core SMc02774 onto two ribose SBPs. Ribose (C_5_H_10_O_5_) and fucose (C_6_H_12_O_5_) are both aldose sugars of very similar structure and hydrogen bond-forming capabilities. The first ribose SBP (PBD: 2DRI; 28% id) from *E. coli* belongs to Class I [31] or cluster B [32] and was crystallised bound to β-D-ribopyranose [36], while the second ribose SBP (PBD: 3KSM; 27% id) from *Hahella chejuensis* was crystallised bound to β-D-ribofuranose [43]. These two ribose-binding SBPs show 23% id to each other. All three SBPs share hydrogen bond-forming amino acids; Arg148 and Gln245 of core SMc02774, connecting to ligand hydroxyl groups on C_1_ and C_2_ in the SBP from *H. chejuensis*, and on C_2_ and C_3_ in that from *E. coli*. Furthermore, two other hydrogen bond-forming amino acids, Lys10 and Asn199 of the core of SMc02774 and assumed to be important for binding L-fucose, are found in closely matching position in both ribose SBPs.

### FRET biosensor for β-diglucosides

This biosensor is based on SMc04259, a SBP of an ABC-transporter of the CUT1 sub-family. The gene for SMc04259 was induced up to 19-fold by gentiobiose [9]. Contiguous with the ABC transporter operon (SMc04256-9) is a gene annotated as encoding a putative β-mannosidase (SMc04255, ManB) [42]. β-mannosidases hydrolyse the terminal, non-reducing end of β-D-mannose residues. Given this predicted enzyme activity it may be that the *in vivo* ligand for this transport system is β1→4 glucopyranosyl-D-mannose, a disaccharide sub-unit of glucomannan, found in plant cell walls.

Purified SMc04259-CY displayed an *in vitro* ligand concentration-dependent increase in FRET ratio of 2.1±0.2% upon binding cellobiose (glc β1→4 glc) (K_d_ 3.3±0.9×10^−8^ M) and gentiobiose (glc β1→6 glc) (K_d_ 5.3±2.1×10^−8^ M) (Table 1, Fig. 2C). There was no change in FRET with maltose (glc α1→4 glc), with the β-disaccharides lactulose (gal β1→4 fru) and lactose (gal β1→4 glc), or with glucose, mannose and xylose (monosaccharides). Unfortunately β1→4 glucopyranosyl-D-mannose, a candidate for the *in vivo* ligand of SMc04259, was not available commercially and we were unable to test our hypothesis. The results obtained show that SMc04259 binding is specific, not only for a di-glucoside, but also for β-linkage, which can be either β1→4 or β1→6, between the subunits. SMc04259-RO displayed a FRET signal, but no change in FRET ratio was detected on binding either cellobiose or gentiobiose.

SWISS-MODEL threads the tertiary structure of SMc04259 on a glucose/galactose binding protein (GGBP) from *Thermus thermophilus* (PDB: 2B3B/2B3F; 23% id) [44]. The monosaccharide-binding GGBP has been classified in Class I [31] and cluster B, SBPs with a hinge region made of three loops positioning the N- and C-termini in opposite domains [32]. All the amino acids forming hydrogen bonds with β-D-galactopyranose and β-D-glucopyranose in GGBP are highly conserved in the disaccharide-binding SMc04259, except for His348 of GGBP whose side chain forms a hydrogen bond with the hydroxyl group of β-C_1_ of galactose and glucose. For the ligand of SMc04259, this hydroxyl group is replaced in cellobiose by the (β1→4) link to the second D-glucopyranose unit and in gentiobiose by the (β1→6) link to the second D-glucopyranose unit.

Of the fifty-eight *S. meliloti* genes cloned in pCYS, five (8.6%) formed successful FRET biosensors. Since we began with only the induction profile of the corresponding gene to a range of chemicals [9], some FRET biosensors may have been missed if the true ligand was not tested in the screening. Others may have been missed as their ligand had been co-purified with the protein initially and hence there was no change in FRET signal on addition of ligand as the protein waas already in the closed (bound) form. For some SBPs there is additional information available, including, in some cases identification of specific ligand(s). This is true of the two biosensors described below; one based on a SBP from *R. leguminosarum* detecting D-galactose and the other detecting dicarboxylates using a protein from *R. capsulatus*.

### D-galactose FRET biosensor

RL2376, a SBP of an ABC-transporter of the CUT2 sub-family, shows 68% id with the D-galactose SBP YtfQ from *E. coli* for which a crystal structure and precise ligand has been determined [28]. In microarray experiments with *R. leguminosarum*, genes encoding the ABC-transporter (RL2376-9) were induced up to 3.6-fold by D-arabinose and up to 2.4-fold by D-galactose when compared with cells grown on glucose [24].

Purified RL2376-CY displayed an *in vitro* ligand concentration-dependent increase in FRET ratio of 5.2±0.4% on binding D-galactose (K_d_ 5.3±1.7×10^−6^ M) (Table 1, Fig. 2B). There was no change in FRET ratio on addition of D-arabinose or D-glucose. The K_d_ value for D-galactose obtained is high, but is in line with the K_d_ for D-galactose (1.7±0.3 µM) of YtfQ from *E. coli* [28]. SWISS-MODEL used YtfQ (PDB: 2VK2) as a template for threading RL2376 core. Given the high amino acid identity (68%) between these two SBPs, it is not surprising that all the hydrogen bond-forming amino acids are conserved in RL2376 (Glu13; Arg17; Asp90; Arg91; Arg145; Asn198 and Asp226), as are Trp16 and Phe169 which provide hydrophobic surfaces packing against the nonpolar portions of the sugar. YtfQ was crystallised only bound to furanose forms of D-galactose [28], and if FBS#D-galactose is similar, it is likely to specifically detect α/β-D-galactofuranoses. Furanoses represent only 7.7% of the four cyclic isomers of D-galactose in solution (two of them with a pyranose (six-membered) ring and two with a furanose (five-membered) ring). We can estimate that the K_d_ for D-galactofuranose specifically would be 408±131 nM if an hypothetical solution of pure D-galactofuranose was used. RL2376-RO displayed a FRET signal but no change in FRET ratio was detected on D-galactose binding.

### C4-dicarboxylate FRET biosensor

The protein encoded by *R. capsulatus* rcc03024 (DctP), a TRAP-transporter SBP, has been characterised as a C4-dicarboxylate binding protein [21], [22], [23]. The role, as well as the distribution and function of TRAP systems, has recently been reviewed [7], [8]. Recombinant purified rcc03024-CY protein displayed an *in vitro* ligand concentration-dependent change in FRET ratio of −2.7±0.2% on binding C4-dicarboxylates succinate (K_d_ 3.6±0.7×10^−8^ M), fumarate (K_d_ 5.8±1.2×10^−8^ M), L-malate (K_d_ 7.0±1.4×10^−8^ M). It bound oxaloacetate (K_d_ 1.6±0.6×10^−5^ M) at a lower affinity (Table 1, Fig. 2D). This much lower affinity for oxaloacetate may reflect that this very unstable dicarboxylic acid is rarely encountered in the environment. There was no change in FRET ratio on addition of maleate (*cis*-isomer of fumarate), malonate (C3), pyruvate (C3), α-ketoglutarate (C5) or citrate (C6).

Two dicarboxylate SBPs from *Bordetella pertussis*, DctP6 (PDB: 2PFZ) and DctP7 (PDB: 2PFY) bound to pyroglutamic acid have been crystallised and their tertiary structure elucidated [45]. RCAP_rcc03024 (DctP) has 25% and 27% id respectively with these proteins but they share only one hydrogen bond-forming amino acid, Arg146. An arginine residue is found in a similar position in two other structurally defined SBPs; SiaP from *H. influenzae* bound to Neu5Ac (PDB: 3B50) [29], [30] and TakP from *Rhodobacter sphaeroides* bound to pyruvate (PDB: 2HZL) [46]. In all cases the side chain of this arginine residue forms two hydrogen bonds with the carboxyl group of their respective substrate [7]. SWISS-MODEL threaded the tertiary structure of DctP on SiaP (27% id). This predicts N- and C-termini occur on the same lobe of the SBP, and classifies it as Class II [31] and cluster E [32]. Recombinant protein rcc03024-RO displayed a FRET signal, but no change in FRET ratio was detected upon binding to L-malate or succinate.

### Conclusions

The ability to use FRET biosensors based on orange/red fluorescence, alongside cyan/yellow FRET is a step towards the detection of multiple metabolites in combination. Shifting to longer wave-lengths for FRET should permit deeper penetration of samples at lower energy, bringing better resolution with lower background auto-fluorescence [48]. The two FRET vectors developed, pCYS and pROS, allow convenient and efficient cloning of SBP encoding genes by In-fusion PCR cloning. To our knowledge, the FRET biosensors for D-quinic acid and C4-dicarboxylates are the first based upon SBPs from TRAP transporters. After characterization of a FRET construct, various techniques, such as circular permutation of ligand-binding domains and optimization of length and sequence of fluorescent proteins to SBP linkers, can be used to improve the change in FRET ratio on ligand binding [11], [25], [49]. The FRET biosensors described in this paper could be developed for study (both *in vivo* and *in vitro*) of four new classes of compounds: cyclic polyols; L-deoxy sugars; β-linked disaccharides and dicarboxylates. Since dicarboxylates are provided by legumes to rhizobial microsymbionts located in root nodule as their carbon and energy source for N_2_-fixation [47] the C4-dicarboxylate FRET biosensor could be developed for *in vivo* spatial and temporal monitoring during formation of legume nodules and during N_2_-fixation. However, in this study our aim has been to identify SBPs that give good FRET signals, but they need considerable customisation and optimisation of signal strength to be used *in vivo*.

## Materials and Methods

### Construction of pCYS

To construct new FRET vector pCYS (for CFP/YFP sensor), primers pr0411/pr0412 (Table S1) were used to amplify by PCR using pRSET FLIPglu-600μΔ11 Aphrodite (Addgene: plasmid 13569) as template (Fig. 1A). This plasmid was derived from pRSET FLIPglu-600μΔ11 [11], [13]. These primers added a SpeI restriction site at both ends of the PCR product. After SpeI digestion of the PCR product, it was ligated, re-forming the SpeI site which was subsequently used for cloning SBP genes between those encoding eCFP and Aphrodite (Fig. 1B).

### Construction of pROS

In order to make a RFP/OFP FRET vector we initially constructed a plasmid with genes for RFP and YFP. This was done by replacing the gene for eCFP in a pCYS clone, with a gene encoding RFP. To excise the gene for eCFP, pLMB291 (SMc02324 cloned in pCYS, Table 1) was digested with BamHI and KpnI. This was ligated using T4 ligase with the gene encoding mKate2 (Evrogen) [27] (RFP), PCR amplified with primers pr0972/pr0973 (Table S1) and digested with both BamHI and KpnI. This re-forms BamHI and KpnI sites at the 5′-and 3′-ends respectively of DNA encoding mKate2. The plasmid constructed, pLMB634 (Table S1), encodes SMc02324 cloned between genes for for RFP and YFP.

In the next step in making a RFP/OFP FRET vector, the YFP of pLMB634 was replaced by OFP. The gene encoding Aphrodite (YFP) was excised from pLMB634 by XhoI/HindIII double restriction digest. The gene for mOrange2 (Clontech) [26] (OFP) was PCR amplified with primers pr1043b/pr1044b (Table S1), and inserted by In-fusion Advantage PCR cloning (Clontech) into XhoI/HindIII digested pLMB634. The XhoI and HindIII sites were re-formed at the 5′- and 3′-ends respectively of mOrange2 yielding pLMB523 (Table 1). DNA encoding the core of SMc02324 (L-rhamnose binding protein) was excised from pLMB523 by SpeI endonuclease digest and re-ligated, yielding pROS (for RFP/OFP sensor). Vector pROS has a single SpeI site between genes encoding mKate2 and mOrange2 (Fig. 1C).

### Cloning of SBP genes

In order to clone only the nucleotides encoding the mature (or core) SBPs, the location of signal peptide cleavage sites in amino acid sequences of SBPs were predicted using SignalP 3.0 and TatP 1.0 (Table S2) [50], [51]. Nucleotide sequences encoding mature SBP genes lacking both N-terminal periplasmic leader sequence and the stop codon were amplified by PCR from chromosomal DNA using a pair of primers (Table S1) each carrying a 16 bp tail identical to the sequence either side of the unique SpeI site of pCYS or pROS. PCR fragments were then inserted into the vector according to the manufacturer's instructions for In-fusion cloning. Correct insertions were identified by PCR amplification using primers prCYSfor/prCYSrev or prROSfor/prROSrev (Table S1), before being confirmed by sequencing.

### Expression and purification of FRET biosensors

Plasmids containing cloned core SBPs were transformed into *E. coli* Rosetta2 (Novagen) by heat shock and grown overnight at 37°C. Auto Induction Medium (Formedium) was inoculated by a 1/10 dilution from the over-night culture, incubated for 2 hours at 30°C (250 rpm) and then for 2 days in the dark at 17°C. Cells were harvested by centrifugation, resuspended in 50 mM Tris-HCl, pH 8.5, and disrupted by ultrasonication. FRET biosensor proteins were purified by nickel affinity chromatography. Samples were loaded to a 1 ml HisTrap FF column at 4°C on an AKTA Basic 10 (GE Life Sciences), the column was rinsed with 50 mM Tris-HCl containing 10 mM imidazole, then step-eluted with 50 mM Tris-HCl containing 100 mM imidazole at pH 8.5. Eluates were dialysed twice in 50 mM Tris-HCl, pH 8.5 then stored at 4°C in the dark.

### In vitro characterisation of FRET biosensors

Ligand-induced FRET ratio changes of purified FRET biosensor were screened using a LS55 luminescence spectrometer (PerkinElmer Instruments). Excitation (ex.) was at (λ/slit width) 433/10 nm (CFP) or 549/10 nm (OFP). Emission (em.) was measured at 475/10 nm (CFP), 527/10 nm (YFP), 573/10 nm (OFP) and 644/10 nm (RFP). To confirm that Aphrodite and mKate2 were properly folded their fluorescence was individually tested by direct excitation at 510/6 nm and 588/10 nm respectively.

Affinity constants (K_d_) of FRET biosensors with specific ligands were established using a monochromator microplate reader (Safire, Tecan, Switzerland; CFP ex.: (λ\bandwidth) 433/12 nm, em.: 475/12 nm; YFP em.: 527/12 nm. OFP ex.: 533/12 nm, em.: 563/12 nm; RFP em.: 615/12 nm). Proteins were diluted to give relative fluorescence units (RFU) in the detection range of 30,000 to 50,000 RFU at a gain of 90 to 100, and then were added to a dilution series (10^−3^ to 10^−11^ M) of ligand in Tris-HCl (pH 8.5, 50 mM). The change in relative emission intensities at 527/477 nm (YFP/CFP) or 615/563 nm (RFP/OFP) was used to determine K_d_ by fitting the titration curves to a single-site-binding isotherm [52]:
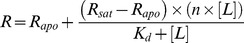
with: R, ratio; R_apo_, ratio without ligand; R_sat_, ratio at saturation of ligand; *n*, number of equal binding sites; [L], ligand concentration. Non-linear regression was used to fit the curves using SigmaPlot 11 (Systat Software Inc.) (Hill equation, 4 Parameters, b = 1). All measurements were done in quadruplicate.

Protein structures were threaded using SWISS-MODEL [53], [54], [55] to allow an approximation of the structure to be determined. This is useful for determining the likely type I or type II folding of the proteins and allows rational consideration of future modification to protein structures.

## Supporting Information

Table S1 **Primers used in this study.**
(DOC)

Table S2 **Summary of SBPs cloned in pCYS.**
(XLSX)
