# Supplementary material for: Mining the Sinorhizobium meliloti Transportome to Develop FRET Biosensors for Sugars, Dicarboxylates and Cyclic Polyols
Source: PLoS One. 2012 Sep 24;7(9):e43578. doi: 10.1371/journal.pone.0043578 (PMC3454389; doi:10.1371/journal.pone.0043578)
Supplement: Table S1 — Primers used in this study. (DOC) [file pone.0043578.s001.doc]

**Table S1. Primers used in this study.**

| **Vector constructed/Gene cloned (plasmid)^1^** | **Primer pair** | primer length | **Description (binds to - RE sites contained – used for- orientation)^2^** | **Primer sequence^3^** |
| --- | --- | --- | --- | --- |
| pCYS | pr0411 | 30 | pFLIPglu600µΔ11 Aphrodite - **SpeI** - T4 ligation after SpeI digest - Rev | AAAA**ACTAGT**GGTACCGATCCCGGCGGCGG |
| (cyan/yellow) | pr0412 | 29 | pFLIPglu600µΔ11 Aphrodite - **SpeI** - T4 ligation after SpeI digest - For | TTTT**ACTAGT**CTCGAGTTGTTTACGGGCG |
|  | prCYSfor | 20 | pCYS - screening and sequencing constructs - For | GCACCCAGTCCGCCCTGAGC |
|  | prCYSrev | 20 | pCYS - screening and sequencing constructs - Rev | CTTATGCCCGTTAACATCCC |
| SMc02324 | pr0972 | 36 | mKate2 - **BamHI** – T4 ligation with pCYS digested BamHI/KpnI - For | AA**GGATCC**GGGCCGCATGGTGAGCGAGCTGATTAAG |
| (pLM634) | pr0973 | 35 | mKate2 - **KpnI** – T4 ligation with pCYS digested BamHI/KpnI - Rev | TTCACTAGT**GGTACC**TCTGTGCCCCAGTTTGCTAG |
| (red/yellow) |  |  |  |  |
| SMc02324 | pr1043b | 37 | mOrange - **XhoI** - BD cloning with Xho\HindIII cut pLMB634 - For | CTTCACTAGT**CTCGAG**ATGGTGAGCAAGGGCGAGGAG |
| (pLMB523) | pr1044b | 38 | mOrange - **HindIII** - BD cloning with Xho\HindIII cut pLMB634 - Rev | CAGCCGGATC**AAGCTT**TCACTTGTACAGCTCGTCCATG |
|  | prROSfor | 20 | pROS - screening and sequencing constructs - For | GCCAGATACTGCGACCTCCC |
| (red/orange) | prROSrev | 21 | pROS - screening and sequencing constructs - Rev | GATGGCCATGTTATTCTCCTC |
| *mglB* | pr0606 | 35 | MglB core from pFLIPglu600µΔ13 - **SpeI** - BD cloning in pCYS - For | GATCGGTACC**ACTAGT**GCTGATACTCGCATTGGTG |
| (pLMB240) | pr0607 | 37 | MglB core from pFLIPglu600µΔ13 - **SpeI** - BD cloning in pCYS - Rev | ACAACTCGAG**ACTAGT**AGCCAGGTTGTCTTTATCTAC |
| SMa0067 | pr0636 | 37 | core SMa0067 - **SpeI** - BD cloning in pCYS - For | GATCGGTACC**ACTAGT**GAAGGTCTCAGCATCGCCTTC |
|  | pr0637 | 37 | core SMa0067 - **SpeI** - BD cloning in pCYS - Rev | ACAACTCGAG**ACTAGT**GCGCAGACCCTGCTTCGCCAG |
| SMa0157 | pr0576 | 36 | core SMa0157 - **SpeI** - BD cloning in pCYS - For | GATCGGTACC**ACTAGT**CAGACGGTGTTGAAGGCGTC |
|  | pr0577 | 37 | core SMa0157 - **SpeI** - BD cloning in pCYS - Rev | ACAACTCGAG**ACTAGT**CTTCACGGCGAGCGCCTTTTC |
| SMa0203 | pr0646 | 37 | core SMa0203 - **SpeI** - BD cloning in pCYS - For | GATCGGTACC**ACTAGT**GACACCTCGGCCAAGAAGATC |
|  | pr0647 | 35 | core SMa0203 - **SpeI** - BD cloning in pCYS - Rev | ACAACTCGAG**ACTAGT**CTTTGCCGACTCGATGACC |
| SMa0252 | pr0632 | 37 | core SMa0252 - **SpeI** - BD cloning in pCYS - For | GATCGGTACC**ACTAGT**GAGTTCAACGACCGCAACATC |
|  | pr0633 | 35 | core SMa0252 - **SpeI** - BD cloning in pCYS - Rev | ACAACTCGAG**ACTAGT**CTTGCGGATCTCTTCCAGC |
| SMa0799 | pr0578 | 36 | core SMa0799 - **SpeI** - BD cloning in pCYS - For | GATCGGTACC**ACTAGT**GCCGATCTCGTTTTCACGAG |
|  | pr0579 | 36 | core SMa0799 - **SpeI** - BD cloning in pCYS - Rev | ACAACTCGAG**ACTAGT**CTTCGCCTGCCAGGCGTACC |
| SMa1427 | pr0674 | 37 | core SMa1427 - **SpeI** - BD cloning in pCYS - For | GATCGGTACC**ACTAGT**GCATTGACGGATCCCGCGATC |
|  | pr0675 | 36 | core SMa1427 - **SpeI** - BD cloning in pCYS - Rev | ACAACTCGAG**ACTAGT**GGACCGATACTGAATTTGTC |
| SMa2129 | pr0580 | 37 | core SMa2129 - **SpeI** - BD cloning in pCYS - For | GATCGGTACC**ACTAGT**GGGCCAACCTCACTGAAGATC |
|  | pr0581 | 37 | core SMa2129 - **SpeI** - BD cloning in pCYS - Rev | ACAACTCGAG**ACTAGT**CTTCACCTCCGCGGTATTGAG |
| SMa2305 | pr0648 | 37 | core SMa2305 - **SpeI** - BD cloning in pCYS - For | GATCGGTACC**ACTAGT**CAATCGAGCGAGGTGACGATC |
|  | pr0649 | 35 | core SMa2305 - **SpeI** - BD cloning in pCYS - Rev | ACAACTCGAG**ACTAGT**GAGCTTGAAGATCCGCCGGC |
| **SMb20036** | pr0582 | 37 | core SMb20036 - **SpeI** - BD cloning in pCYS - For | GATCGGTACC**ACTAGT**GAGATCCGCGATCAGACGGTC |
| (pLMB229) | pr0583 | 35 | core SMb20036 - **SpeI** - BD cloning in pCYS - Rev | ACAACTCGAG**ACTAGT**TTGGCCGCGGGCGGTGCCG |
| (pLMB521) | pr1075 | 35 | core SMb20036 - **SpeI** - BD cloning in pROS - For | CAGAGGTACC**ACTAGT**GAGATCCGCGATCAGACGG |
|  | pr1076 | 35 | core SMb20036 - **SpeI** - BD cloning in pROS - Rev | CCATCTCGAG**ACTAGT**TTGGCCGCGGGCGGTGCCG |
| SMb20072 | pr0598 | 37 | core SMb20072 - **SpeI** - BD cloning in pCYS - For | GATCGGTACC**ACTAGT**GAGAACGTCGGCATCACCATC |
|  | pr0599 | 35 | core SMb20072 - **SpeI** - BD cloning in pCYS - Rev | ACAACTCGAG**ACTAGT**CTGATTTGCGGTAGCGTAG |
| SMb20144 | pr0736 | 38 | core SMb20144 - **SpeI** - BD cloning in pCYS - For | GATCGGTACC**ACTAGT**GCATCCGGGCCGGTCAAGATCG |
|  | pr0737 | 38 | core SMb20144 - **SpeI** - BD cloning in pCYS - Rev | ACAACTCGAG**ACTAGT**TTTGATCTTGATCTCGGAGAGC |
| SMb20295 | pr0584 | 36 | core SMb20295 - **SpeI** - BD cloning in pCYS - For | GATCGGTACC**ACTAGT**CAAGAGGCGCGCACATTGCG |
|  | pr0585 | 35 | core SMb20295 - **SpeI** - BD cloning in pCYS - Rev | ACAACTCGAG**ACTAGT**ACGGAGATCGAGCAGCGTC |
| SMb20316 | pr0660 | 37 | core SMb20316 - **SpeI** - BD cloning in pCYS - For | GATCGGTACC**ACTAGT**CAGGACGCTCCCTCCGTCGTG |
|  | pr0661 | 35 | core SMb20316 - **SpeI** - BD cloning in pCYS - Rev | ACAACTCGAG**ACTAGT**GAACGGGTACTGGCTGTAG |
| SMb20325 | pr0652 | 36 | core SMb20325 - **SpeI** - BD cloning in pCYS - For | GATCGGTACC**ACTAGT**GCCGAATTGTCTATGGCGGC |
|  | pr0653 | 37 | core SMb20325 - **SpeI** - BD cloning in pCYS - Rev | ACAACTCGAG**ACTAGT**CCAAGCGTCGCCCTTGAGTTC |
| SMb20410 | pr0642 | 37 | core SMb20410 - **SpeI** - BD cloning in pCYS - For | GATCGGTACC**ACTAGT**GCTGAGCCGGAGAGTTGCAAG |
|  | pr0643 | 37 | core SMb20410 - **SpeI** - BD cloning in pCYS - Rev | ACAACTCGAG**ACTAGT**GCTTTTCAACGCGCTCTCAAC |
| SMb20428 | pr0678 | 37 | core SMb20428 - **SpeI** - BD cloning in pCYS - For | GATCGGTACC**ACTAGT**GACGAGAACAAGCTCGAGGAG |
|  | pr0679 | 38 | core SMb20428 - **SpeI** - BD cloning in pCYS - Rev | ACAACTCGAG**ACTAGT**TTTCGCGGCGCAGAGCTTTTCG |
| SMb20442 | pr0630 | 36 | core SMb20442 - **SpeI** - BD cloning in pCYS - For | GATCGGTACC**ACTAGT**CAGACGGCGCTCAAATGGGC |
|  | pr0631 | 37 | core SMb20442 - **SpeI** - BD cloning in pCYS - Rev | ACAACTCGAG**ACTAGT**CTTGATGGCGCGGATAGCCTC |
| SMb20570 | pr0724 | 37 | core SMb20570 - **SpeI** - BD cloning in pCYS - For | GATCGGTACC**ACTAGT**GAAGCTCCGGAACCGGTGACG |
|  | pr0725 | 36 | core SMb20570 - **SpeI** - BD cloning in pCYS - Rev | ACAACTCGAG**ACTAGT**ATTCGCCGCGGCGGACGTCG |
| **SMb20712** | pr0662 | 37 | core SMb20712 - **SpeI** - BD cloning in pCYS - For | GATCGGTACC**ACTAGT**GAGACTATCGGCGTGTCGATG |
| (pLMB319) | pr0663 | 37 | core SMb20712 - **SpeI** - BD cloning in pCYS - Rev | ACAACTCGAG**ACTAGT**GTTCTTCGTCTGATATTGCGC |
| SMb20856 | pr0634 | 37 | core SMb20856 - **SpeI** - BD cloning in pCYS - For | GATCGGTACC**ACTAGT**GGCATCGCAAACGCCGCCGAC |
|  | pr0635 | 34 | core SMb20856 - **SpeI** - BD cloning in pCYS - Rev | ACAACTCGAG**ACTAGT**GAAAACGGCGTCGGCGCG |
| SMb20902 | pr0644 | 35 | core SMb20902 - **SpeI** - BD cloning in pCYS - For | GATCGGTACC**ACTAGT**AAGGATCTGGTCATCGGCG |
|  | pr0645 | 36 | core SMb20902 - **SpeI** - BD cloning in pCYS - Rev | ACAACTCGAG**ACTAGT**TTTGCAGGCAGCAACGTCGC |
| SMb20931 | pr0656 | 37 | core SMb20931 - **SpeI** - BD cloning in pCYS - For | GATCGGTACC**ACTAGT**CAGAAGAAACAGCTCGTCATC |
|  | pr0657 | 36 | core SMb20931 - **SpeI** - BD cloning in pCYS - Rev | ACAACTCGAG**ACTAGT**TTTCTGGATGCAGGTATCCG |
| SMb21097 | pr0690 | 37 | core SMb21097 - **SpeI** - BD cloning in pCYS - For | GATCGGTACC**ACTAGT**GCCGGCCTGCCCGAGCGGATC |
|  | pr0691 | 37 | core SMb21097 - **SpeI** - BD cloning in pCYS - Rev | ACAACTCGAG**ACTAGT**CTTCACCGCTTCGCCATTGAC |
| SMb21103 | pr0626 | 37 | core SMb21103 - **SpeI** - BD cloning in pCYS - For | GATCGGTACC**ACTAGT**GCGGACCTGCCCGGCAAGTTC |
|  | pr0627 | 37 | core SMb21103 - **SpeI** - BD cloning in pCYS - Rev | ACAACTCGAG**ACTAGT**GAGCTTGCCGGCGTCTTCGAG |
| SMb21133 | pr0586 | 37 | core SMb21133 - **SpeI** - BD cloning in pCYS - For | GATCGGTACC**ACTAGT**GACACGACCATCCTGAACGTG |
|  | pr0587 | 37 | core SMb21133 - **SpeI** - BD cloning in pCYS - Rev | ACAACTCGAG**ACTAGT**TTGCCCCGGTCGGTAGATCTG |
| SMb21135 | pr0588 | 37 | core SMb21135 - **SpeI** - BD cloning in pCYS - For | GATCGGTACC**ACTAGT**CAACAGGCATCGAGCAAGCTC |
|  | pr0589 | 36 | core SMb21135 - **SpeI** - BD cloning in pCYS - Rev | ACAACTCGAG**ACTAGT**CTTGTACTCGATGGGAAAAC |
| SMb21151 | pr0672 | 36 | core SMb21151 - **SpeI** - BD cloning in pCYS - For | GATCGGTACC**ACTAGT**GCGGACCAGGAAATCAGCTG |
|  | pr0673 | 36 | core SMb21151 - **SpeI** - BD cloning in pCYS - Rev | ACAACTCGAG**ACTAGT**CTTGTACTGTTCGAGGTCGG |
| SMb21221 | pr0670 | 37 | core SMb21221 - **SpeI** - BD cloning in pCYS - For | GATCGGTACC**ACTAGT**GAAACGACGCTGAACGCGCTG |
|  | pr0671 | 36 | core SMb21221 - **SpeI** - BD cloning in pCYS - Rev | ACAACTCGAG**ACTAGT**GAGACCGCTATTCTCGGCGG |
| SMb21273 | pr0602 | 37 | core SMb21273 - **SpeI** - BD cloning in pCYS - For | GATCGGTACC**ACTAGT**CAGGAGAAGACCCTTACCATC |
|  | pr0603 | 36 | core SMb21273 - **SpeI** - BD cloning in pCYS - Rev | ACAACTCGAG**ACTAGT**CTGGCCGACCTTGGCGTTCC |
| SMb21345 | pr0638 | 37 | core SMb21345 - **SpeI** - BD cloning in pCYS - For | GATCGGTACC**ACTAGT**GAACTGCCAAAACTGGCGCAG |
|  | pr0639 | 36 | core SMb21345 - **SpeI** - BD cloning in pCYS - Rev | ACAACTCGAG**ACTAGT**GTAGGCGTTGGCGAGTTCGG |
| SMb21353 | pr0732 | 36 | core SMb21353 - **SpeI** - BD cloning in pCYS - For | GATCGGTACC**ACTAGT**CAGACGGTGCTGCGCTCGTC |
|  | pr0733 | 37 | core SMb21353 - **SpeI** - BD cloning in pCYS - Rev | ACAACTCGAG**ACTAGT**CTGTACTGCGCGTACGTCCTC |
| SMb21377 | pr0666 | 37 | core SMb21377 - **SpeI** - BD cloning in pCYS - For | GATCGGTACC**ACTAGT**GACGGCATCGGCGCATCGCTC |
|  | pr0667 | 36 | core SMb21377 - **SpeI** - BD cloning in pCYS - Rev | ACAACTCGAG**ACTAGT**TTTCCCGGTATAGACGCCCG |
| SMb21421 | pr0640 | 36 | core SMb21421 - **SpeI** - BD cloning in pCYS - For | GATCGGTACC**ACTAGT**GCGGAGATCAATCTGGACGC |
|  | pr0641 | 38 | core SMb21421 - **SpeI** - BD cloning in pCYS - Rev | ACAACTCGAG**ACTAGT**TTTGCAGGCAAACTCCGGATAG |
| SMb21526 | pr0590 | 36 | core SMb21526 - **SpeI** - BD cloning in pCYS - For | GATCGGTACC**ACTAGT**GAAACCAGCGTCGTCGTGGG |
|  | pr0591 | 38 | core SMb21526 - **SpeI** - BD cloning in pCYS - Rev | ACAACTCGAG**ACTAGT**CTTGGCGGCTTCTTTCGCATAG |
| SMb21587 | pr0628 | 39 | core SMb21587 - **SpeI** - BD cloning in pCYS - For | GATCGGTACC**ACTAGT**GCCGAACTCGTCGTCGGCTTTTC |
|  | pr0629 | 41 | core SMb21587 - **SpeI** - BD cloning in pCYS - Rev | ACAACTCGAG**ACTAGT**GTAGCCGAGGCCTTTCTTTTCTTCG |
| SMb21647 | pr0604 | 36 | core SMb21647 - **SpeI** - BD cloning in pCYS - For | GATCGGTACC**ACTAGT**TCCGAGCCGACCGTGGTGCC |
|  | pr0605 | 36 | core SMb21647 - **SpeI** - BD cloning in pCYS - Rev | ACAACTCGAG**ACTAGT**ATTGATCGGGCCGCTTTCGC |
| SMb21652 | pr0658 | 37 | core SMb21652 - **SpeI** - BD cloning in pCYS - For | GATCGGTACC**ACTAGT**AAGGAAATCACCATCTGGTGC |
|  | pr0659 | 37 | core SMb21652 - **SpeI** - BD cloning in pCYS - Rev | ACAACTCGAG**ACTAGT**CTGGATCTGTGCGCTGACCTC |
| SMc00265 | pr0730 | 35 | core SMc00265 - **SpeI** - BD cloning in pCYS - For | GATCGGTACC**ACTAGT**GCGGACTTCACCTATAAGC |
|  | pr0731 | 35 | core SMc00265 - **SpeI** - BD cloning in pCYS - Rev | ACAACTCGAG**ACTAGT**GCCGAGGCTGGTGCCTACG |
| SMc00672 | pr0680 | 37 | core SMc00672 - **SpeI** - BD cloning in pCYS - For | GATCGGTACC**ACTAGT**TCCTATTGCGGCGACGGCAAG |
|  | pr0681 | 38 | core SMc00672 - **SpeI** - BD cloning in pCYS - Rev | ACAACTCGAG**ACTAGT**CTTGAGACCGGCTTCGATCTTG |
| SMc01496 | pr0668 | 37 | core SMc01496 - **SpeI** - BD cloning in pCYS - For | GATCGGTACC**ACTAGT**GAGACCCTGACCATTGCCACC |
|  | pr0669 | 36 | core SMc01496 - **SpeI** - BD cloning in pCYS - Rev | ACAACTCGAG**ACTAGT**CTTGATGTAGCCGGCCTTGG |
| SMc01628 | pr0664 | 36 | core SMc01628 - **SpeI** - BD cloning in pCYS - For | GATCGGTACC**ACTAGT**GCCGACTTCGACTGGAAGGC |
|  | pr0665 | 35 | core SMc01628 - **SpeI** - BD cloning in pCYS - Rev | ACAACTCGAG**ACTAGT**GCCGAGCCCGGCTTCCGCG |
| SMc01652 | pr0728 | 37 | core SMc01652 - **SpeI** - BD cloning in pCYS - For | GATCGGTACC**ACTAGT**CAGGAGCCGCTGAAAGAGCTC |
|  | pr0729 | 35 | core SMc01652 - **SpeI** - BD cloning in pCYS - Rev | ACAACTCGAG**ACTAGT**CCGCCCGCCGATCACACCG |
| SMc01827 | pr0592 | 37 | core SMc01827 - **SpeI** - BD cloning in pCYS - For | GATCGGTACC**ACTAGT**GCCGAAAAGGTCACACTTCAG |
|  | pr0593 | 35 | core SMc01827 - **SpeI** - BD cloning in pCYS - Rev | ACAACTCGAG**ACTAGT**CTTAAGCGCCGCGTCCGTG |
| SMc02021 | pr0738 | 38 | core SMc02021 - **SpeI** - BD cloning in pCYS - For | GATCGGTACC**ACTAGT**ACGGGCGCAAAGCGGAAAGTCG |
|  | pr0739 | 38 | core SMc02021 - **SpeI** - BD cloning in pCYS - Rev | ACAACTCGAG**ACTAGT**GCCGAAGCGCGCCTCGCGCTCG |
| SMc02171 | pr0594 | 35 | core SMc02171 - **SpeI** - BD cloning in pCYS - For | GATCGGTACC**ACTAGT**GCCGAGGTGTCGGCATGCC |
|  | pr0595 | 36 | core SMc02171 - **SpeI** - BD cloning in pCYS - Rev | ACAACTCGAG**ACTAGT**GCCCCAGCACTTCTCCATGC |
| **SMc02324** | pr0622 | 35 | core SMc02324 - **SpeI** - BD cloning in pCYS - For | GATCGGTACC**ACTAGT**GAAAACAAGAAGATCGCGC |
| (pLMB291) | pr0623 | 35 | core SMc02324 - **SpeI** - BD cloning in pCYS - Rev | ACAACTCGAG**ACTAGT**GAAGATCTTCGCGAATTCC |
| SMc02344 | pr0726 | 35 | core SMc02344 - **SpeI** - BD cloning in pCYS - For | GATCGGTACC**ACTAGT**GGCGAGGCGAAGCCGGACG |
|  | pr0727 | 38 | core SMc02344 - **SpeI** - BD cloning in pCYS - Rev | ACAACTCGAG**ACTAGT**GTTGCTGGCGGCAAGTCTTTTG |
| SMc02415 | pr0720 | 36 | core SMc02415 - **SpeI** - BD cloning in pCYS - For | GATCGGTACC**ACTAGT**GCGACGCTGCGTTGGGGCAG |
|  | pr0721 | 36 | core SMc02415 - **SpeI** - BD cloning in pCYS - Rev | ACAACTCGAG**ACTAGT**TTCGGCCATTTGCGTGTGCC |
| SMc02509 | pr0740 | 37 | core SMc02509 - **SpeI** - BD cloning in pCYS - For | GATCGGTACC**ACTAGT**GCGGAAAAGCTCAAGGCCGTC |
|  | pr0741 | 38 | core SMc02509 - **SpeI** - BD cloning in pCYS - Rev | ACAACTCGAG**ACTAGT**TTGCGACAAGCCTTTCGCGATC |
| SMc02514 | pr0676 | 37 | core SMc02514 - **SpeI** - BD cloning in pCYS - For | GATCGGTACC**ACTAGT**GGGATGGACGAGGCAAAGCAG |
|  | pr0677 | 37 | core SMc02514 - **SpeI** - BD cloning in pCYS - Rev | ACAACTCGAG**ACTAGT**GTTCGTCTGCCAGCTCTTGAC |
| SMc02737 | pr0722 | 36 | core SMc02737 - **SpeI** - BD cloning in pCYS - For | GATCGGTACC**ACTAGT**GCCGAGCCGGAAAGCTGCGG |
|  | pr0723 | 37 | core SMc02737 - **SpeI** - BD cloning in pCYS - Rev | ACAACTCGAG**ACTAGT**GAGGCCGAGGGCGGCCTTGAC |
| **SMc02774** | pr0624 | 37 | core SMc02774 - **SpeI** - BD cloning in pCYS - For | GATCGGTACC**ACTAGT**CAGGACGTCACGATTCCCATC |
| (pLMB292) | pr0625 | 37 | core SMc02774 - **SpeI** - BD cloning in pCYS - Rev | ACAACTCGAG**ACTAGT**GTTGACCTTCGGATTCAGAAG |
| SMc02832 | pr0704 | 37 | core SMc02832 - **SpeI** - BD cloning in pCYS - For | GATCGGTACC**ACTAGT**GAGGAACAACCCGTCTGGCAC |
|  | pr0705 | 38 | core SMc02832 - **SpeI** - BD cloning in pCYS - Rev | ACAACTCGAG**ACTAGT**TTTTGCAGCCGTGTTTTTCGAC |
| SMc02873 | pr0781 | 37 | core SMc02873 - **SpeI** - BD cloning in pCYS - For | GATCGGTACC**ACTAGT**CAGGACGCAACGCTGACGATC |
|  | pr0782 | 36 | core SMc02873 - **SpeI** - BD cloning in pCYS - Rev | ACAACTCGAG**ACTAGT**TTTTACCGGCTTGAACCAGC |
| SMc03061 | pr0654 | 37 | core SMc03061 - **SpeI** - BD cloning in pCYS - For | GATCGGTACC**ACTAGT**GCCGACCTGAAATTCAAGCCG |
|  | pr0655 | 38 | core SMc03061 - **SpeI** - BD cloning in pCYS - Rev | ACAACTCGAG**ACTAGT**CTTGATGGAGTCCCATGCCTTC |
| SMc03196 | pr0744 | 37 | core SMc03196 - **SpeI** - BD cloning in pCYS - For | GATCGGTACC**ACTAGT**GCGGAGAAGGTCACCGTGTTC |
|  | pr0745 | 35 | core SMc03196 - **SpeI** - BD cloning in pCYS - Rev | ACAACTCGAG**ACTAGT**TTCCAGAACCGTGAAGCCC |
| **SMc04259** | pr0600 | 35 | core SMc04259 - **SpeI** - BD cloning in pCYS - For | GATCGGTACC**ACTAGT**ACCGATCTCGAGGTCACGC |
| (pLMB345) | pr0601 | 35 | core SMc04259 - **SpeI** - BD cloning in pCYS - Rev | ACAACTCGAG**ACTAGT**ATCCGCAGCGGCGATGATG |
| (pLMB522) | pr1073 | 35 | core SMc04259 - **SpeI** - BD cloning in pROS - For | CAGAGGTACC**ACTAGT**ACCGATCTCGAGGTCACGC |
|  | pr1074 | 35 | core SMc04259 - **SpeI** - BD cloning in pROS - Rev | CCATCTCGAG**ACTAGT**ATCCGCAGCGGCGATGATG |
| SMc04396 | pr0596 | 37 | core SMc04396 - **SpeI** - BD cloning in pCYS - For | GATCGGTACC**ACTAGT**GCCGAGAATGTGGAAGTACTG |
|  | pr0597 | 36 | core SMc04396 - **SpeI** - BD cloning in pCYS - Rev | ACAACTCGAG**ACTAGT**CTTCGCAGCCTCGACCGCCG |
| **RCAP_** | pr0849 | 37 | core DctP - **SpeI** - BD cloning in pCYS - For | GATCGGTACC**ACTAGT**GAGCCCATCGTGATCAAGTTC |
| **rcc03024** | pr0850 | 37 | core DctP - **SpeI** - BD cloning in pCYS - Rev | ACAACTCGAG**ACTAGT**TTCCGCCGTCGCGGCCTTGAC |
| (pLMB414) | pr1081 | 35 | core DctP - **SpeI** - BD cloning in pROS - For | CAGAGGTACC**ACTAGT**GAGCCCATCGTGATCAAGT |
| (pLMB520) | pr1082 | 35 | core DctP - **SpeI** - BD cloning in pROS - Rev | CCATCTCGAG**ACTAGT**TTCCGCCGTCGCGGCCTTG |
| RL2204 | pr0570 | 36 | core RL2204 - **SpeI** - BD cloning in pCYS - For | GATCGGTACC**ACTAGT**CCACCACTCTGTCAGACGTC |
|  | pr0571 | 37 | core RL2204 - **SpeI** - BD cloning in pCYS - Rev | ACAACTCGAG**ACTAGT**ACGAACCGGCGGTGCGTACTG |
|  | pr1083 | 35 | core RL2204 - **SpeI** - BD cloning in pROS - For | CAGAGGTACC**ACTAGT**ACCACTCTGTCAGACGTCA |
|  | pr1084 | 35 | core RL2204 - **SpeI** - BD cloning in pROS - Rev | CCATCTCGAG**ACTAGT**ACGAACCGGCGGTGCGTAC |
| **RL2376** | pr0843 | 37 | core RL2376 - **SpeI** - BD cloning in pCYS - For | GATCGGTACC**ACTAGT**GCCGGCCTCACTGTCGGCTTC |
| (pLMB394) | pr0844 | 37 | core RL2376 - **SpeI** - BD cloning in pCYS - Rev | ACAACTCGAG**ACTAGT**GTAGCCCTGACCCTTCTTCTC |
| (pLMB526) | pr1079 | 35 | core RL2376 - **SpeI** - BD cloning in pROS - For | CAGAGGTACC**ACTAGT**GCCGGCCTCACTGTCGGCT |
|  | pr1080 | 35 | core RL2376 - **SpeI** - BD cloning in pROS - Rev | CCATCTCGAG**ACTAGT**GTAGCCCTGACCCTTCTTC |
| RL2721 | pr0845 | 37 | core RL2721 - **SpeI** - BD cloning in pCYS - For | GATCGGTACC**ACTAGT**GCGGAACTGAAGAAGCTCGGC |
|  | pr0846 | 37 | core RL2721 - **SpeI** - BD cloning in pCYS - Rev | ACAACTCGAG**ACTAGT**CGGATGGTTGGCGATGAAGCC |
| RL3540 | pr0572 | 37 | core RL3540 - **SpeI** - BD cloning in pCYS - For | GATCGGTACC**ACTAGT**GATATCACCATCGGCCTGATC |
|  | pr0573 | 36 | core RL3540 - **SpeI** - BD cloning in pCYS - Rev | ACAACTCGAG**ACTAGT**CTCGGCGGCGACGATCTTGC |
| RL3617 | pr0841 | 36 | core RL3617 - **SpeI** - BD cloning in pCYS - For | GATCGGTACC**ACTAGT**CAGGACAAGGGGCTTGTCGG |
|  | pr0842 | 35 | core RL3617 - **SpeI** - BD cloning in pCYS - Rev | ACAACTCGAG**ACTAGT**CTTCAGCTTGTCTTCGGTG |
| RL3745 | pr0574 | 36 | core RL3745 - **SpeI** - BD cloning in pCYS - For | GATCGGTACC**ACTAGT**GACGTTCTCATCGCTGTCGC |
|  | pr0575 | 36 | core RL3745 - **SpeI** - BD cloning in pCYS - Rev | ACAACTCGAG**ACTAGT**CATGCCCTGCGGGAAGTAGC |
|  | pr1085 | 35 | core RL3745 - **SpeI** - BD cloning in pROS - For | CAGAGGTACC**ACTAGT**GACGTTCTCATCGCTGTCG |
|  | pr1086 | 35 | core RL3745 - **SpeI** - BD cloning in pROS - Rev | CCATCTCGAG**ACTAGT**CATGCCCTGCGGGAAGTAG |
| pRL80060 | pr0847 | 39 | core pRL80060 - **SpeI** - BD cloning in pCYS - For | GATCGGTACC**ACTAGT**GCGGAGGTTCCGGTCACGGAATC |
|  | pr0848 | 39 | core pRL80060 - **SpeI** - BD cloning in pCYS - Rev | ACAACTCGAG**ACTAGT**TTCGGCAATCTGATCGACAGAGG |
| pRL110400 | pr0835 | 37 | core pRL110400 - **SpeI** - BD cloning in pCYS - For | GATCGGTACC**ACTAGT**CAGAGCGCGACCGACGGCAAG |
|  | pr0836 | 36 | core pRL110400 - **SpeI** - BD cloning in pCYS - Rev | ACAACTCGAG**ACTAGT**CTTCACCAGATCGCAGCCGC |
| pRL120500 | pr0839 | 37 | core pRL120500 - **SpeI** - BD cloning in pCYS - For | GATCGGTACC**ACTAGT**GCCCCGGCCGTGCTGGCCCAG |
|  | pr0840 | 37 | core pRL120500 - **SpeI** - BD cloning in pCYS - Rev | ACAACTCGAG**ACTAGT**TTTTCCGAGGTTCAATTTTCC |
| pRL120593 | pr1031 | 36 | core pRL120593 - **SpeI** - BD cloning in pCYS -For | GATCGGTACC**ACTAGT**CAGGAACTGACCATCTTCTG |
|  | pr1032 | 36 | core pRL120593 - **SpeI** - BD cloning in pCYS -Rev | ACAACTCGAG**ACTAGT**CTTGTGGCGTCCGTATTTCG |

^1^ Successful FRET biosensors are shown in bold and plasmid names are shown in brackets, Fluorescent protein pairs for vectors are indicated.

^2^ Restriction enzyme sites are shown in bold. ^3^ Restriction enzyme sites are highlighted in yellow.
